# Obstructive lithiasis of the lower bile duct discovered four decades after cholecystectomy and its management by ideal choledochotomy: a case report

**DOI:** 10.1186/s13256-023-04052-3

**Published:** 2023-07-24

**Authors:** Joël Igor Kamla, Guy Aristide Bang, Joel Noutakdie Tochie, Landry Wakheu Tchuenkam, Arthur Georges Essomba

**Affiliations:** 1 Department of Surgery, Sangmelima Reference Hospital, Sangmelima, Cameroon; 2 Faculty of Medicine and Pharmaceuticals Sciences, University of Ebolowa, Ebolowa, Cameroon; 3 Faculty of Medicine and Biomedical Sciences, University of Yaounde I, Yaoundé, Cameroon; 4 Department of Surgery, Yaounde University Hospital Center, Yaoundé, Cameroon; 5 Anaesthesiology and Critical Care Units, Douala Laquintinie Hospital, Douala, Cameroon; 6 Department of Surgery, Adlucem Hospital, Douala, Cameroon

**Keywords:** Choledocholithiasis, Laparotomy, Choledochotomy, Biliary drainage

## Abstract

**Background:**

Residual lithiasis is the presence of stones in the common bile duct, ignored after one or more biliary interventions. We report an atypical case of chronic symptomatic lithiasis of the lower bile duct occurring 41 years after biliary surgery, managed successfully by ideal choledochotomy.

**Case presentation:**

A 68-year-old Black African female with several past laparotomies including a cholecystectomy forty-one years ago presented with hepatic colic-type pain that had been intermittent for several years but worsened recently. Her clinical, biological, and imaging test assessments were suggestive of a residual obstructive lithiasis of the lower common bile duct. Through an open right subcostal laparotomy approach, a dilated bile duct of approximately 3 cm was found and managed by transverse choledochotomy in which the stone was extracted in retrograde manner. After confirmation of disobstruction, a primitive bile duct suture without biliary drainage was performed and a tubular drain was positioned under the liver. The postoperative course was uneventful at follow-up of 30 days.

**Conclusion:**

Residual choledocholithiasis can be avoided. We performed an ideal choledochotomy, of which the follow-up was simple.

## Background

Cholelithiasis is the most common biliary tract pathology in the world, affecting approximately 5–25% of adults [1, 2]. In Africa, its frequency is underestimated [3]. The gallbladder is by far the most frequent location and is complicated in 10–20% of cases of lithiasis of the common bile duct (LCBD) [1]. LCBD is certainly secondary to the migration of a cholelithiasis in more than 85% of cases, but it can be primitive, either following a stasis upstream of a cicatricial stenosis post cholecystectomy, or of intrahepatic origin as is prevalent in Asia [4, 5]. It can also be residual or recurrent [4, 6]. Residual lithiasis is defined by the presence of stones in the common bile duct, ignored after one or more biliary interventions [1]. Its clinical and biological polymorphism poses a diagnostic dilemma. Endoscopic treatment is currently the first line in the management of residual LCBD, but prevention by intraoperative cholangiography during the initial intervention remains the gold standard of care [1, 5, 7, 8]. We report herein a case of symptomatic choledocholithiasis after initial cholecystectomy four decades ago and managed by ideal choledochotomy.

## Case presentation

A 68-year-old Cameroonian was admitted to our emergency room for right hypochondrium pain of 2-week duration without fever. She had had a cholecystectomy 41 years previously, indicated for symptomatic cholelithiasis. A few months after this cholecystectomy, she began to experience a succession of increasingly painful, intermittent colicky, and self-limiting episodes of right hypochondrium pain. This pain was localized, aggravated by deep inspiration, triggered by high-fat meals, and had no relieving factor. Her past history was also relevant for four median laparotomies for acute appendicitis, intestinal obstruction, gastroduodenal perforation, and total hysterectomy.

**Fig. 1:**
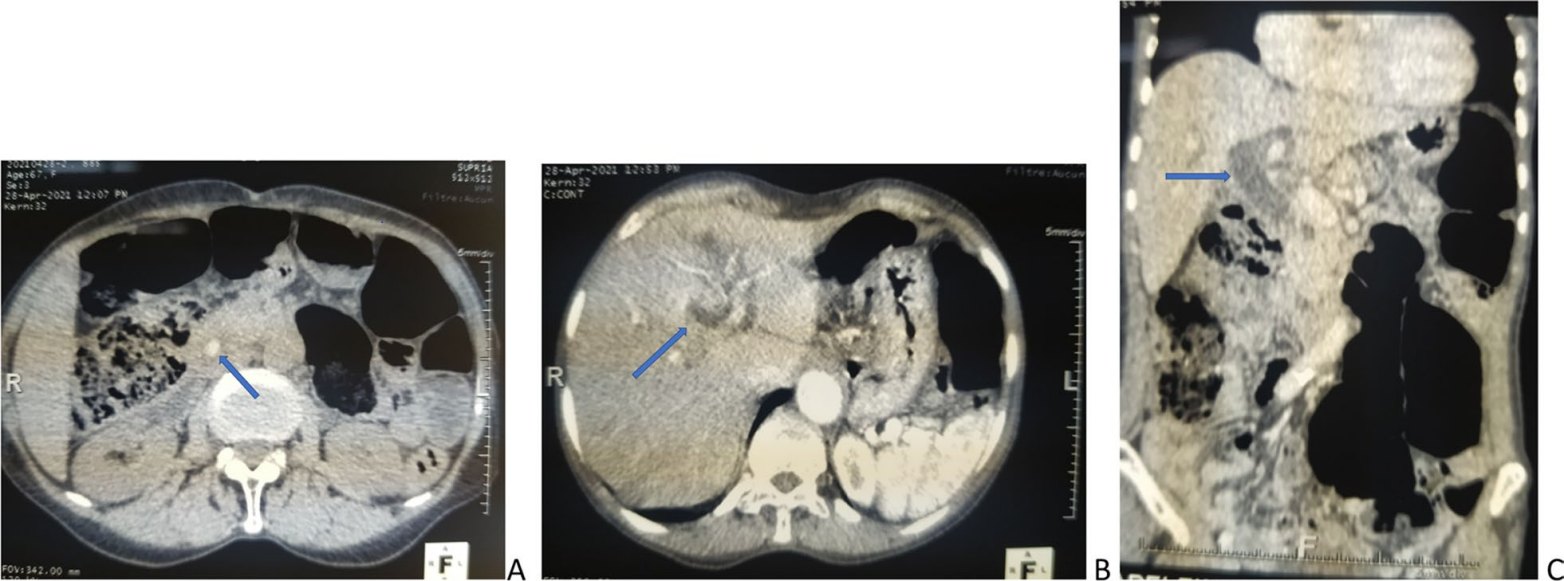
Abdominal scan. **A** Lower common bile lithiasis measuring 12 × 8 mm (blue arrow). **B** Dilation of the intrahepatic bile ducts (blue arrow). **C** Dilatation of the common bile duct (blue arrow)

On clinical assessment, she had subicteric sclera, noticeable right hypochondrial tenderness induced by deep palpation, and fecal discoloration. There was no weight loss, hepatomegaly, or palpable abdominal mass. A laboratory panel showed a cholestasis syndrome (serum alkaline phosphatase increased 1.5-fold and conjugated bilirubin by 5-fold), hepatic cytolysis syndrome (alanine aminotransferase 27-fold increased and aspartate aminotransferase 11-fold increased), and lipasemia 1.7 times normal. The septic screen was negative. An abdominal computed tomography (CT) scan (Fig. 1) showed an isolated lower common bile stone (12 × 8 mm) with dilatation of the bile ducts and no enhancement of their walls. Faced with this prolonged biliary colic-type pain associated with disturbance of hepatopancreatic tests, the working diagnosis was residual obstructive and symptomatic lithiasis of the lower common bile duct (CBD).

We adopted a medicosurgical management plan. Initially, she was hyperhydrated and placed on antibiotic therapy (ofloxacin and metronidazole). Given the unavailability of endoscopic techniques in our resource-constrained context and in view of the history of laparotomy which could make laparoscopy difficult, we opted for choledochotomy by laparotomy.

**Fig. 2:**
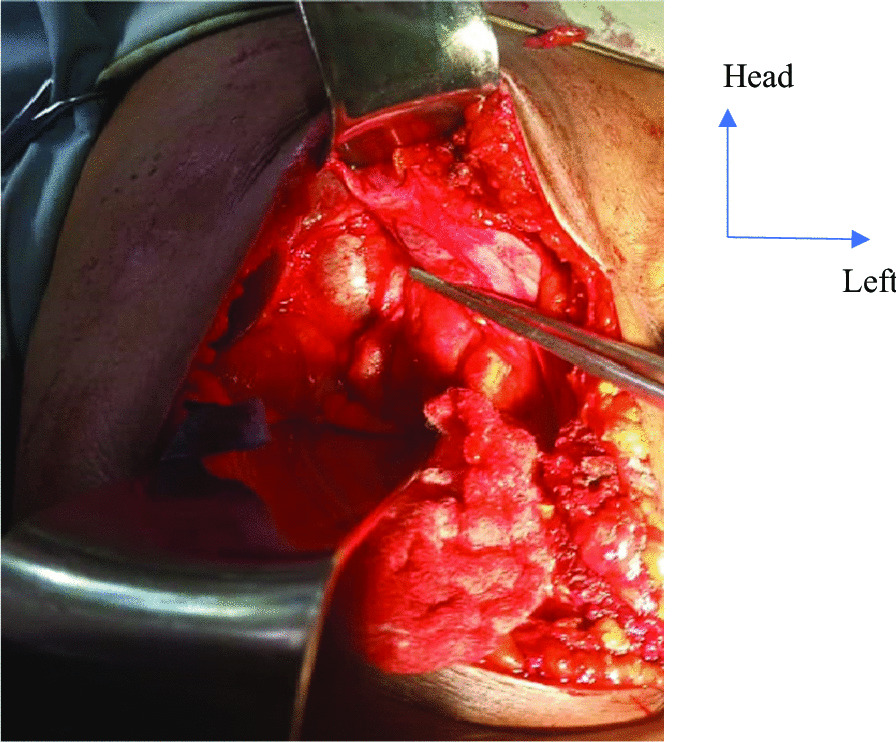
Intraoperative view of the dilated common bile duct

**Fig. 3:**
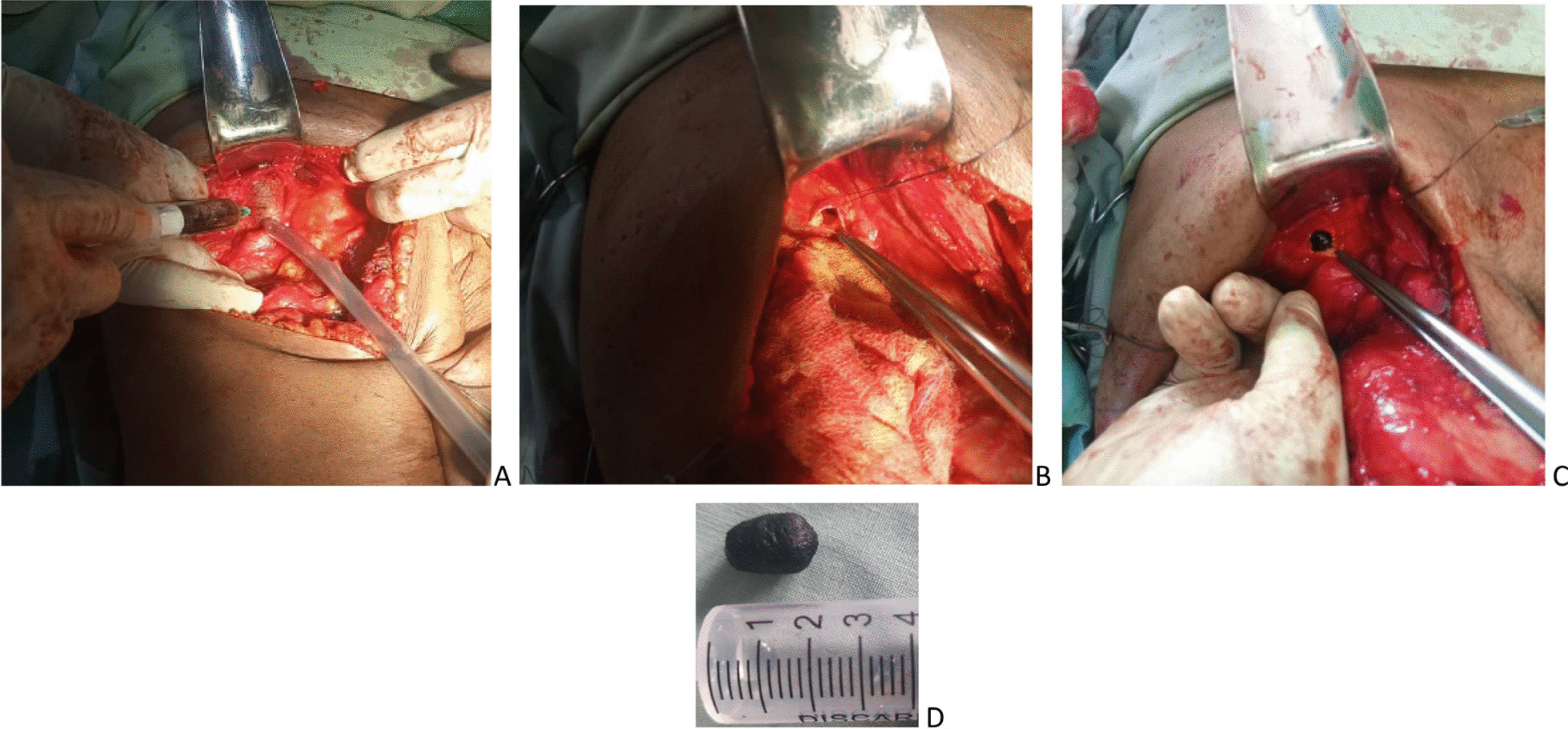
Choledochotomy. **A** Bile puncture returning macroscopically normal bile. **B** Transverse choledochotomy. **C** Extraction of the stone by retrograde finger mobilization. **D** Pigment lithiasis extract

After preanesthetic consultation, the patient was classified as American Society of Anesthesiologists (ASA) III and a laparotomy was performed under general anesthesia plus orotracheal intubation. Intraoperative findings after a right subcostal approach were subhepatic coloepiploic adhesions, a macroscopically normal liver, and a dilated bile duct about 3 cm in diameter (Fig. 2) contrasting with the absence of jaundice. The procedures performed were as follows (Fig. 3): The first stage was the choledochal puncture bringing back a macroscopically normal bile sent for bacteriology. After this puncture, the second step was a transverse choledochotomy of about 1 cm after placement of the presentation sutures on the edges of the incision. The next step was exploration of the common bile duct with a 5-mm rigid choledochoscopy to extract the stone via the Dormia probe. However, this was unsuccessful because the visualization of the stone was not satisfactory. We, therefore, proceeded to a partial duodenopancreatic detachment followed by a retrograde digital expression of the common bile duct, thus allowing extraction of the stone by choledochotomy. The control of the disobstruction was done by choledochoscopy and a nasogastric tube French  10, easily exteriorized in the duodenal lumen. Finally, in view of the complete disobstruction, a dilated bile duct with a noninflammatory wall, and the absence of papillary stenosis, it was decided to perform a so-called ideal bile duct suture using a continuous 4-0 resorbable suture, and a tubular drain was positioned under the liver. The postoperative course was uneventful. Oral feeding and active mobilization were initiated the next day. The drain was removed on the fourth day, and the patient was discharged on the fifth postoperative day. Right hypochondrial pain completely regressed, and the clinical examination as well as hepatopancreatic tests carried out 1 month later were normal.

## Discussion

The exact incidence and prevalence of LCBD are not known, but at time of cholecystectomy, approximately 20% of patients present with LCBD, with the incidence increasing with age [1]. The frequency of residual lithiasis is difficult to estimate, since the patients are not always seen again, nor reoperated by the same surgeon. It varies from 2% to 10% after cholecystectomy [9, 10].

Unlike cholelithiasis, LCBD is rarely asymptomatic and must always be sought, first by collecting predictive factors for LCBD, then, if these are present, by the most sensitive imaging studies (endoscopic ultrasound, biliary magnetic resonance imaging (MRI), and intraoperative cholangiography) [5, 11]. Residual lithiasis of the CBD represents the most frequent cause of “post-cholecystectomy” syndrome [6]. It is a heterogeneous group of symptoms (mainly upper abdominal pain and dyspepsia) that persist or reappear after a cholecystectomy [6]. Another manifestation of LCBD is the migration syndrome. This is an incomplete form without formation of a picture of cholangitis, associated or not with a “pancreatic reaction.” The mechanism, unlike true cholangitis, is a transient impaction of a stone in the papilla, followed either by spontaneous elimination in the gastrointestinal tract by forcing of the papilla, or by opening up of the stone, which becomes mobile again in the bile duct (ball valve effect) [12]. This clinical form was probably the case in our patient.

Treatment modalities for LCBD have changed radically over the past 30 years, following the spectacular spread of imaging, including magnetic resonance cholangiography, endoscopy, and laparoscopy [1]. All LCBD, even asymptomatic, must be treated because of the potential exposure to complications such as cholangitis and acute biliary pancreatitis [13, 14]. In Western and Eastern countries, surgical treatment of LCBD is primarily based on endoscopic biliary sphincterotomy (EBS) [1, 5]. In our resource-constrained African context, laparoscopy coupled with choledochoscopy is possible but remains technically cumbersome. However, the indications for treatment by laparotomy remain numerous, while laparoscopy was obviously going to be technically difficult with high probabilities of conversion to laparotomy owing to the multi-scared abdomen of the index patient with several intraperitoneal adhesion [15]. Thus, the management of our patient should have ideally consisted of endoscopic retrograde cholangiopancreatography followed by EBS, but in the absence of this therapeutic modality in our resource-limited Cameroonian milieu and given the history of supra-mesocolic laparotomy, we opted for an ideal open choledochotomy.

Extraction of the stone by choledochotomy is the technique most often used when there is contraindications or failures of the transcystic route [15]. It is only possible on a bile duct with diameter of at least 8 mm and in the absence of acute inflammatory changes [15–17]. After opening the duct, the disobstruction can be done by retrograde mobilization with a finger, as performed in this patient. However, it can be instrumental by hyperpressure washing with lukewarm serum, using a Dormia probe or stone forceps [15]. In case of lithiasis enclosed in the papilla that resists any extraction, one can resort to contact lithotripsy. In the absence of lithotripsy, postoperative EBS is preferable to transduodenopapillary biliary sphincterotomy [1, 2, 5]. The control of disobstruction should preferably be done by choledochoscopy because intraoperative cholangiography risks causes of error such as air bubbles or absence of duodenal passage after repeated instrumental maneuvers [5]. We used the choledoscope and a nasogastric tube to check the disobstruction. Their easy exteriorization in the duodenal lumen made it possible not only to confirm the disobstruction but also to exclude papillary stenosis, which is a risk factor for primitive lithiasis of CBD. When conditions permit, choledochorraphy will be done without biliary drainage. This “ideal” option was first described in 1917 by Halstead [4], then endorsed by several comparative studies indicating a significant reduction in mortality and morbidity, rendering obsolete the dogma of external biliary drainage, almost systematic after choledochotomy [16, 18, 19]. However, subhepatic drainage is warranted to detect a possible bile leak.

In view of all the above, routine postoperative cholangiography after cholecystectomy may help to reduce the frequency of residual lithiasis, whose management is difficult in underprivileged environments. The postoperative cholangiography avoids other diagnostic means and thus represents an economy of means. It does not prevent accidental lesions of the common bile duct but ensures timely diagnosis and management [7, 8].

## Conclusion

Residual lithiasis of the CBD can be encountered in practice by all surgeons. We performed an ideal choledochotomy by laparotomy without postoperative complications. However, prevention remains the best treatment option, leading to the need for a worldwide practice of intraoperative cholangiography, particularly in low-and middle-income countries in Africa and Asia where cholelithiasis is either underreported or very prevalent.

## Data Availability

Data sharing is not applicable to this article as no datasets were generated or analyzed during the current study.

## Abbreviations

ALAT: Alanine aminotransferase
ASAT: Aspartate aminotransferase
CBD: Common bile duct
EBS: Endoscopic biliary sphincterotomy
LCBD: Lithiasis of the common bile duct
